# Supplementary figures and images for: Efficacy and safety of anti-viral therapy for Hepatitis B virus-associated glomerulonephritis: A meta-analysis
Source: PLoS One. 2020 Jan 15;15(1):e0227532. doi: 10.1371/journal.pone.0227532 (PMC6961902; doi:10.1371/journal.pone.0227532)

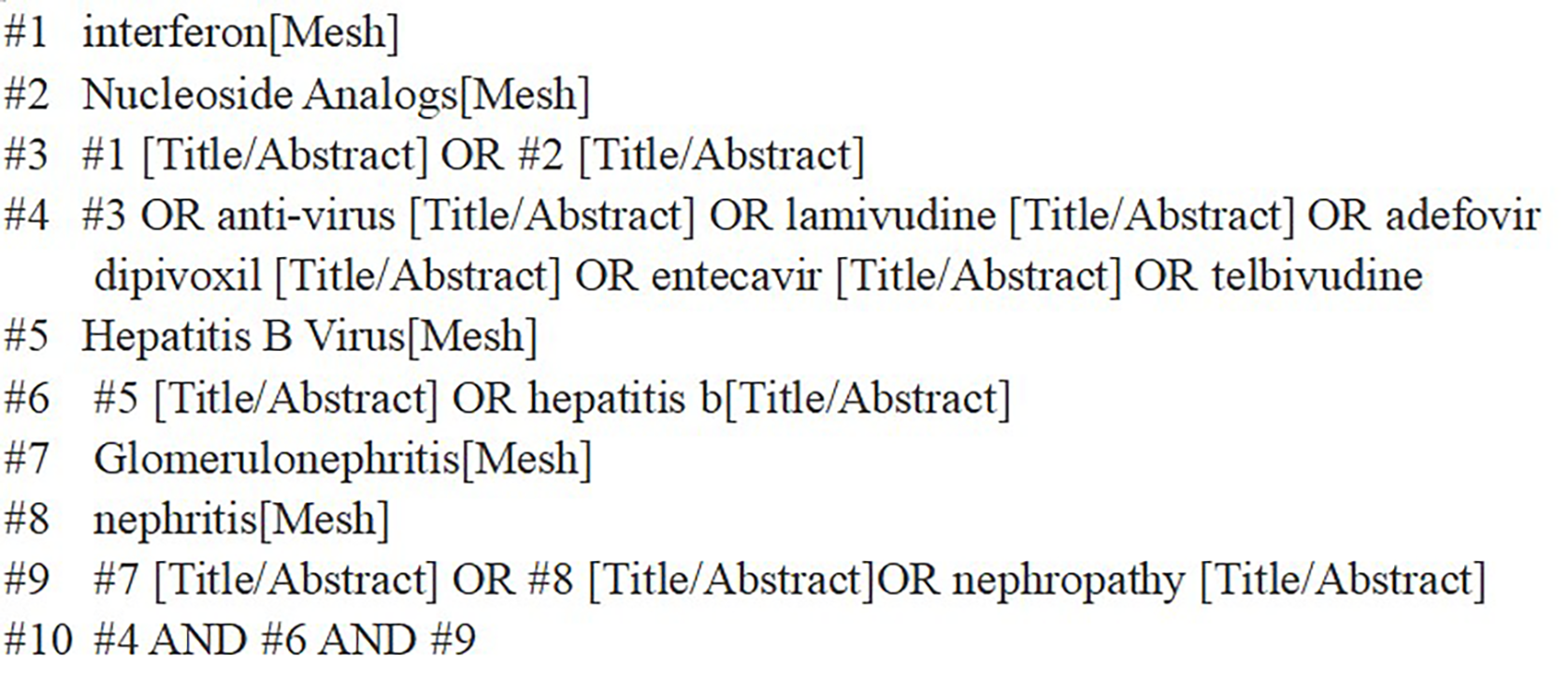

Supplement: S1 Fig — (TIF) [file pone.0227532.s001.tif]

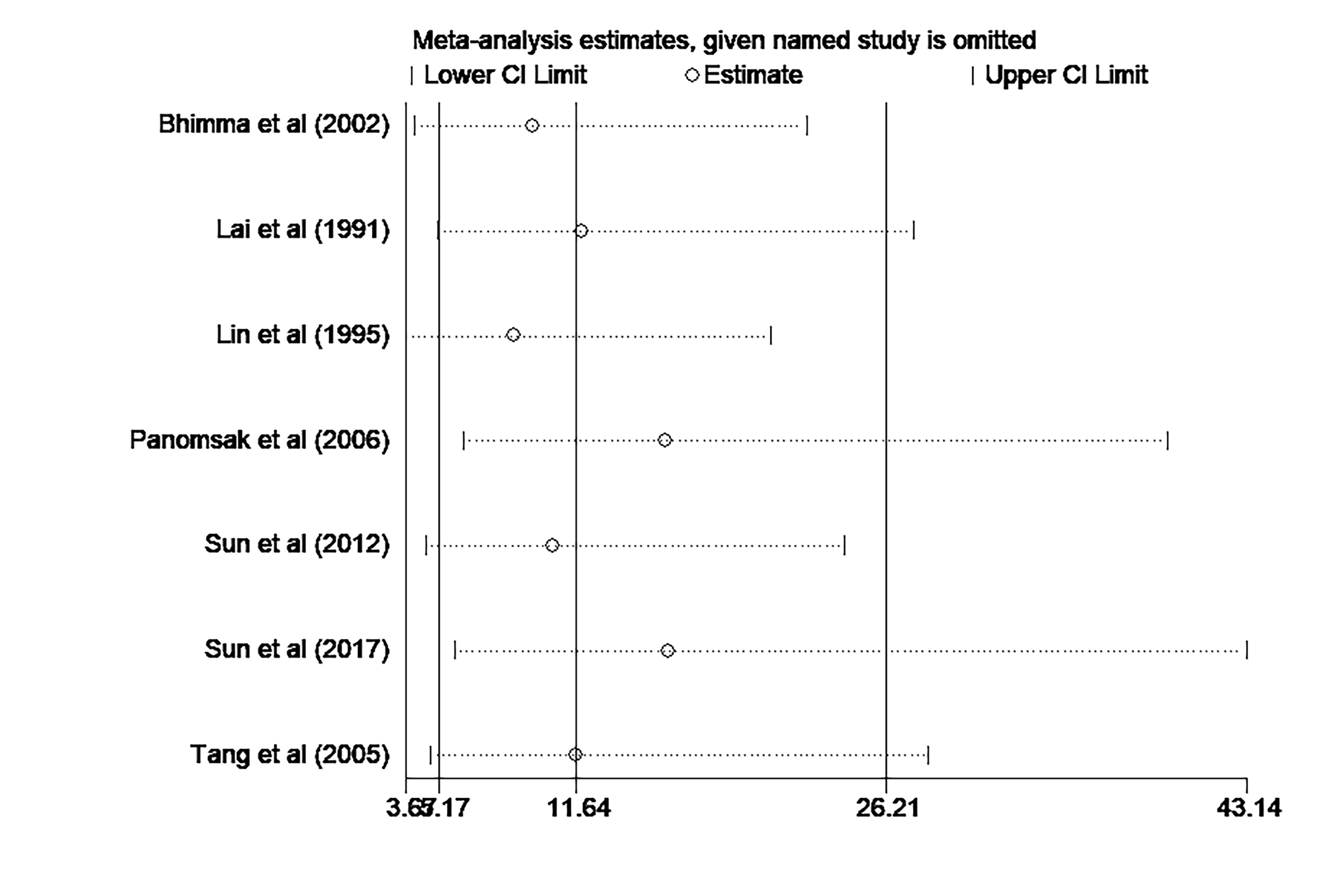

Supplement: S3 Fig — (TIF) [file pone.0227532.s003.tif]

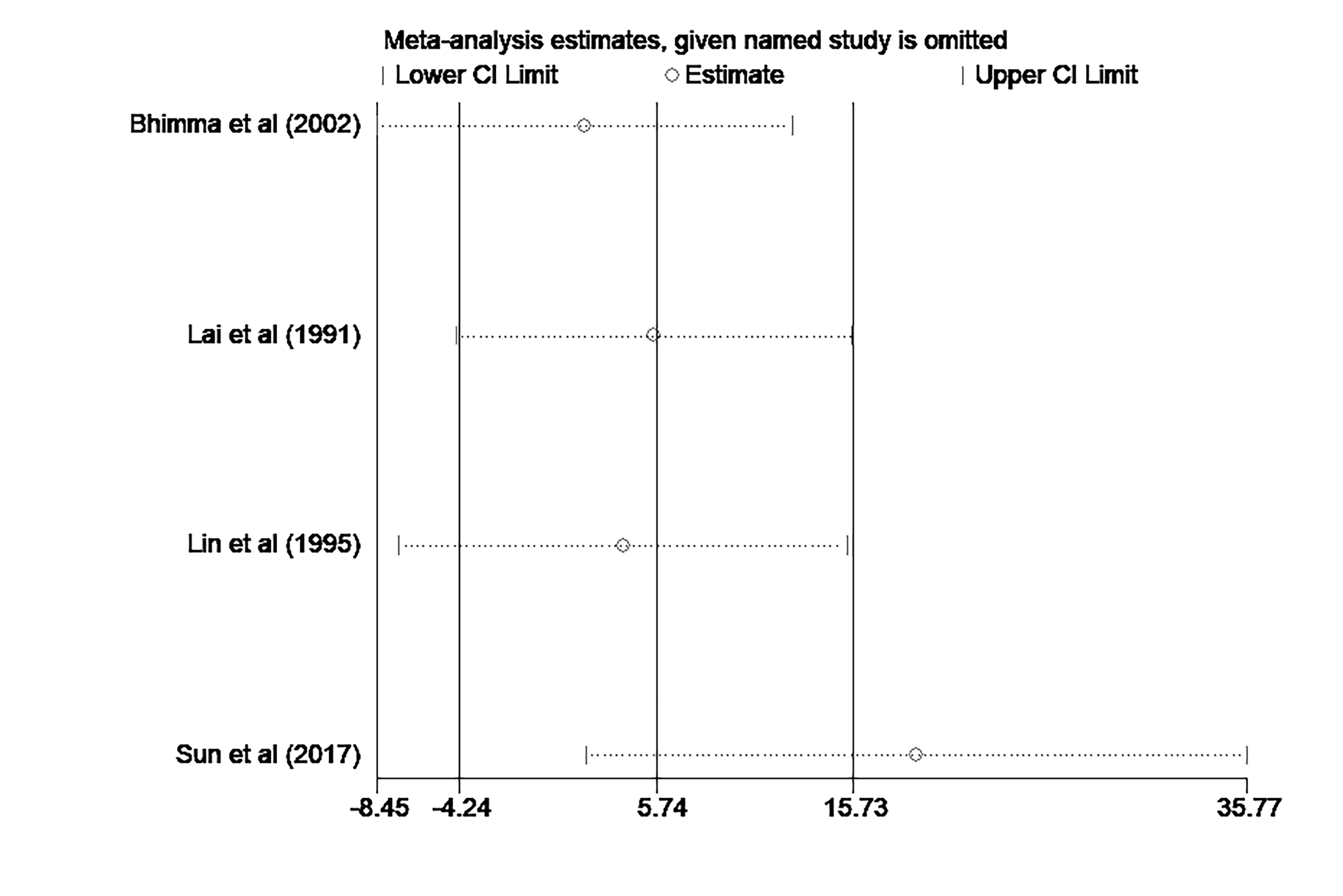

Supplement: S4 Fig — (TIF) [file pone.0227532.s004.tif]

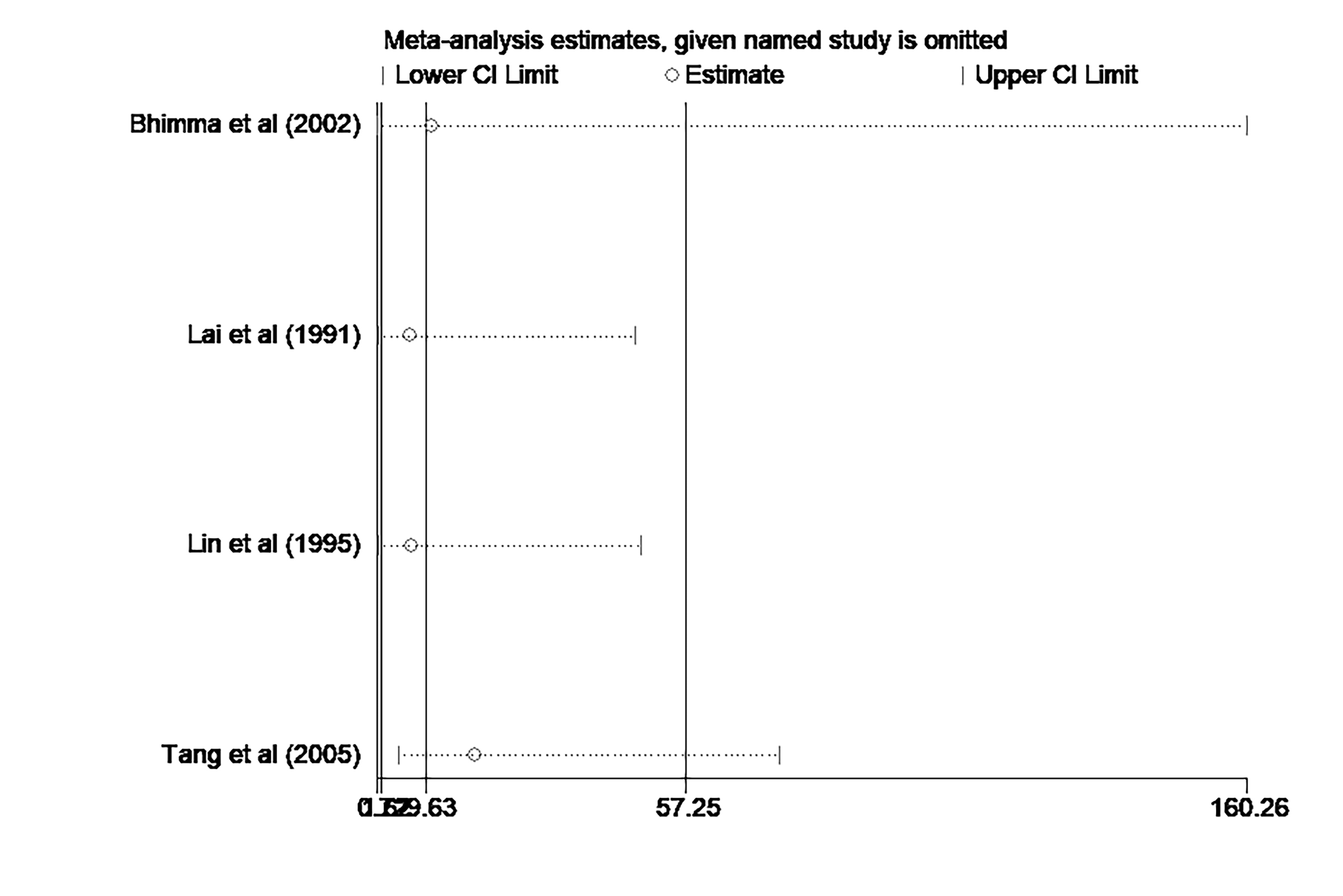

Supplement: S5 Fig — (TIF) [file pone.0227532.s005.tif]
