# Supplementary material for: Efficacy and safety of anti-viral therapy for Hepatitis B virus-associated glomerulonephritis: A meta-analysis
Source: PLoS One. 2020 Jan 15;15(1):e0227532. doi: 10.1371/journal.pone.0227532 (PMC6961902; doi:10.1371/journal.pone.0227532)
Supplement: Attachment — Submitted filename: Response to reviewers.docm [file pone.0227532.s007.docm]

# Response to reviewers

Dear Editors：

Thank you for your letter of comments and for the referee’s comments concerning our manuscript entitled “Efficacy and safety of anti-viral therapy for Hepatitis B virus-associated glomerulonephritis: a meta-analysis”. We have studied all comments carefully and have made corrections. We have send the revised manuscript and revised portion were marked in red. We really hope to meet with your approval.

Comment # 1: Authors used # to show the authors with equal contribution however, only one author's name tagged with #.

Correction: We have made correction according to your comment, two authors “Baohui Fu” and “Yue Ji” were tagged with # to show the authors with equal contribution.

Comment # 2: In the first line of abstract it has been pointed to "corticosteroid" while there is nothing on corticosteroids in the study design and results.

Correction: The article is focus on “anti-viral therapy for Hepatitis B virus-associated glomerulonephritis”, we mentioned "corticosteroid" was inappropriate, so we removed "corticosteroid" from abstract.

Comment #3: In abstract, "proteinuria 18 (OR=10.48, 95% CI: 4.60-23.89, I^2^=0%, P=0.80)". Is the P for heterogeneity or overall effect? It should be clarified.

Correction: It is really true as you suggested, P=0.80 was for heterogeneity in article, but we felt that P=0.80 didn’t have to add after I^2^ statistic, the heterogeneity can be clarified by I²statistic already, so we deleted P=0.80 and P=0.26. We hope our changes can meet with your approval,page2,line 13-14.

Comment # 4: I guess the results for eGFR should be the mean difference of eGFR instead of OR.

Correction: We feel sorry that it was a writing error, we have changed OR into MD, page2, line17.

Comment # 5: In introduction, “where four studies^[4,6,7]^” while 3 study have been cited, page 3, line 20.

Correction: We feel sorry that it was a writing error, we have changed four into three.

Comment # 6: In Methods, “In the absence of statistically significant heterogeneity I^2^>50%”, I^2^ >50% means significant heterogeneity.

Correction: We feel sorry that it was a writing error, we have modified to “In the presence of”, page 6, line 18.

Comment # 7: Page 11, Line 14, "Eight studies were conducted evaluating" 7 or 8?

Correction: We are very sorry for our incorrect writing, we have changed eight into seven, page10, line15.

Comment # 8: Page 12, Line 1, "fixed model" or fixed-effect model?

Correction: We are very sorry for our incorrect writing, we have modified to “fixed effect model”,page 12, line 3.

Comment # 9: Page 12, Line 4, heterogeneity was highly significant (I^2^=0%, *P*=0.80). I^2^=0% and significant heterogeneity?

Correction: We are very sorry for our incorrect writing, we have modified to“heterogeneity was not significant ”, page 11, line 2.

Comment # 10: Page 12, Line 10, “We conducted a sensitivity analysis of all studies (Fig 5).” but 6 studies entered in sensitivity analysis.

Correction: We are very sorry for our negligence, we have replaced a new Fig 5.

Comment # 11： Page 12, Lines 12-15 and in Figure 4, these overall effects caused by repeated inclusion of studies in the meta-analysis. I recommend to exclude it from manuscript and from the Total OR (95% CI) in the bottom part of forest plot in Figure 4. The same can be true for Figure 8 and Figure 9 with inclusion of studies multiple times make a false total overall effect.

Correction: We quite agree with your opinion, so we replaced new Figure 4, Figure 8 and Figure 9. In new figures, we removed repeated total overall effect in the bottom part of forest plots.

Comment #12：Page 14, “Meanwhile, in trials with IFNs, the treatment group was not obviously affected (*P*<0.00001, I^2^=100%). ”Are they talking about heterogeneity? The heterogeneity is high with I^2^=100.

Correction: We feel sorry that it was a writing error, we actually wanted to discuss MD here. we have modified to “the therapeutic effects was not obvious (MD=-44.18, 95% CI: -158.27−69.91)”, page 13, line 12.

Comment #13：For pooled effect of clearance of HBeAg in antiviral therapy, the I^2^=63% and yet using fixed effect model.

Correction: We feel sorry that it was a writing error, we used random effect model in original manuscript which could be shown in Fig11, we have modified to “random effect model”, page 13, line 22.

Comment # 14. The editor suggested that there should be less discussion heterogeneity while it is a technical issue while they should concentrate on overall effect of the pooled analysis. They added some information on the mechanism of HBV-GN, however much is not needed and there is lack of information on how significant is the treatment of HBV-GN and what is the recommendation of authors for treatment of HBV-GN based on the results of meta-analysis. Moreover, in a paragraph, the limitations have been included however, I would like to ask authors to highlight what are the strengths of their study and how it is changing the current knowledge while few meta-analysis have been published even recently (2016).

Correction: Thank you for your suggestions, we totally agree with you. We have rewritten this part according to your suggestions. We wrote discussion based on five aspects as follows:

1.Current treatment status and [necessity](C:/Program%20Files%20(x86)/Youdao/Dict/8.5.3.0/resultui/html/index.html#/javascript:;) of our meta-analysis and our strengths.

2. Mechanism of HBV-GN, but we have shorten this part substantially.

3. Analysis of statistical results, we concentrated on overall effect of the pooled analysis and subgroup difference analysis.

4. Suggestions for treatment in clinical.

5. The limitations.

Comment # 15. In PRISMA flow diagram, "115 records after duplicates removed". Are these 115 were duplicates excluded? It is really confusing in the current drawing.

Correction: We have modified to “115 records were duplicated and removed”.

Comment # 16: There is an additional unnecessary Total effect in Figure 11 can be excluded.

Correction: We have replaced a new Fig 11 without total effect.

Answers to reviewers’ questions were as bellows:

Reviewer #1:

Thanks all reviewers for your comments on our paper. We have  revised our paper according to your comments:

Comment # 1:Page 8 line 11 should be changed from "primary aftermath measures" to "primary outcome measures".
Correction: We have changed from "primary aftermath measures" to "primary outcome measures" which were marked in red, page 2, line 7.

Comment # 2: Additional outcome measure of suppression or clearance of hepatitis B DNA should be added.
Correction: We consider that the measure of clearance of hepatitis B DNA is very important, but this indicator was only mentioned in two of our including studies. and they didn’t provide [concrete](C:/Program%20Files%20(x86)/Youdao/Dict/8.5.3.0/resultui/html/index.html#/javascript:;) [value](C:/Program%20Files%20(x86)/Youdao/Dict/8.5.3.0/resultui/html/index.html#/javascript:;), only described numbers of patients which decreased in serum HBV DNA. Due to limited data, we couldn’t make it as an additional outcome. What’s more, we learned that there was no threshold HBV DNA level associated with HBeAg clearance, the National Institutes of Health Workshop proposed an arbitrary serum HBV DNA level of 10^5^ copies/mL to differentiate chronic hepatitis B from an inactive carrier state, another study claimed beyond that, 33% of patients with HBeAg-negative chronic hepatitis had HBV DNA levels that were persistently above 10^5^ copies/mL. So we think only use HBeAg clearance could also represent immune-active phase of disease transition to the inactive carrier state. When reflect the disease conditions, HBeAg clearence could replace serum HBV DNA to some extent. However, if more clinical studies including this quantitative index , we could accurate estimate the effects of drugs and improve our meta-analysis. We have add discussion on serum HBV DNA in manuscript on page 19.

Comment # 3: The current population estimate there are 292 million people with Hepatitis B today.

Correction: It is really true as you suggested, so we changed it to “292 million people” and the first reference on page 3 line 5-9.

Comment # 4: The “tenofovir” is no capitalized;The letter B should be capitalized when discussing the HBV or hepatitis B; glomerulonephritis does not need to be capitalized.
Correction: We feel sorry that it was a writing error, we have corrected each word individually.

Comment # 5: Adefovir and tenofovir analogues are known nephrotoxins
Did the authors look at nephrotoxicity risk in these manuscripts? And effect on GFR ? this may partially explain the lack of change in GFR?

Correction: It’s quite right as you suggested, We didn't take nephrotoxicity risk into account before, so we add discussion about renal injury risk of Adefovir and tenofovir in our manuscript on page 18.

Responses to reviewer #2:

Comment # 1: There are some minor writing mistakes in the manuscript.

Correction: Thank you for your suggestions, we have read the manuscript carefully to find and correct them.
Comment # 2: There is no need to use thirteen figures to show the results. You can remove some unnecessary figures or move them into supplements section. Figure one in unnecessary you can move it to the supplements. Showing the publication bias in a figure is not necessary.

Correction: We took your advice, we move Figure1 to the supplements, and we used Egger’s test and Begg’s test in publication bias instead on page14.

Comment # 3:There is no need to acknowledge the language editor institute.

Correction:Thank you for your advice and we have removed it from our manuscript.

Responses to reviewer #3: Thank you for your recognition, Thanks very much for your kind work and consideration on publication of our paper.

Thank you very much for giving us an opportunity to revise our manuscript, We have tried our best to revise our manuscript  according to the comments. We would like to thank the referees for their helpful comments and hope that we have now produced a more balance and better account of our work. We trust that the revised manuscript is acceptable for publication. Looking forward to hear from you.

Thank you and best regards.

Yours sincerely,

Baohui Fu and Yue Ji

Corresponding author:

Name: Hongtao Yang

Emile: [tjpdyht@163.com](mailto:tjpdyht@163.com)

.
