# Supplementary material for: Efficacy and safety of anti-viral therapy for Hepatitis B virus-associated glomerulonephritis: A meta-analysis
Source: PLoS One. 2020 Jan 15;15(1):e0227532. doi: 10.1371/journal.pone.0227532 (PMC6961902; doi:10.1371/journal.pone.0227532)
Supplement: Attachment — Submitted filename: Response to Reviewers.doc [file pone.0227532.s008.doc]

# Response to reviewers

Dear Editors：

Thank you for your letter of comments and for the referee’s comments concerning our manuscript entitled “Efficacy and safety of anti-viral therapy for Hepatitis B virus-associated glomerulonephritis: a meta-analysis”. We have studied all comments carefully and have made corrections. We have send the revised manuscript and revised portion were marked in red. We really hope to meet with your approval.

Comment # 1:Conclusion of abstract should be concise.

Response: Thank you for your suggestions, we totally agree with you. We have shorten the conclusion as you suggested.

Comment # 2: Page 3, Line 18, use "nucleos(t)ide analogs (NAs)" instead of "nucleoside analogs (NAs)".

Response: We feel sorry that it was a writing error, we have changed "nucleoside analogs (NAs)"into"nucleos(t)ide analogs (NAs)" on page 3, line 16.

Comment #3: I didn't find S1 Table

Response: We feel sorry that it was a writing error, we have add S1 Table in supporting information.

Comment # 4: It was stated "Heterogeneity between data sources was noted in the CR rate of all trials (I^2^=23%, P=0.26) and CCTs (I^2^=9%, P=0.36)." while based on I^2^ there is no significant heterogeneity between studies. Page 12, Lines 9-11.

Response: We feel sorry for our carelessness. We have revised this sentence as "There was no heterogeneity between data sources in the CR of all trials (I^2^=23%, P=0.26)"on page11,line6-7

Comment # 5: What is "(I^2^=0%<50%, P=0.77)"? Page 12, Line 14

Response: We feel sorry that it was a writing error, we have deleted this sentence"without clinical and statistical heterogeneity (I^2^=0%<50%, P=0.77)".

Comment # 6: Page 12, Lines 11-12, "Our analysis determined that the study by Lin et al^[17]^ was the source of heterogeneity.", based on Figure 4, I expect the study by Fang et al to cause heterogeneity.

Response: We feel sorry that it was a writing error, we have re-making a figure4, and we revised as"Our analysis determined that the study by Sun et al^[17]^ and Panomsak et al^[20]^ was the source of heterogeneity."on page11,line9-11.

Comment # 7: Figure 3, the scale of first plot and the two next plots should be the same. all 0.001-1000 or 0.01-100.

Response: We are very sorry for our incorrect writing, we have made a new Figure 3 according to your suggestion, and because of adjustment of figures, Fiure3 turned out to be Figure 2.

Comment # 8: Regarding the sensitivity analysis plots, why the scales are not aligned with the results of meta-analysis? For example, in figure 4, the OR is 2.58 while I expect an OR 11.64 based on the meta-analysis. This is true for other sensitivity analysis plots as well.

Response: We feel sorry the sensitivity analysis plots were wrong and we made new figure4 ,figure9 and figure11, and put them into supporting information as S3 Fig, S4 Fig , S5 Fig.

Comment # 9: Figure 7, in caption of plot, the OR>1 should favours experimental and OR<1 should favours control.

Response: We feel sorry the figure 7 was incorrect and we replaced a new figure 7, and because of adjustment of figures, Fiure3 turned out to be Figure 5.

Responses to reviewer #2: Eleven figures are not recommended in a paper. You can move 4 other figures (2, 4, 9, 11) to the supplementary materials (supporting information).

Response: We feel great thanks for your professional review work onour article. As you are concerned, there are problems that need to modified . According to your nice suggestions, we have moved 4 other figures (2, 4, 9, 11) to the supporting information.

Responses to reviewer #4:

Comment # 1: The manuscript needs writing edit for English language.

Response: We totally agree with your suggestions, so we send our manuscript to American Journal Experts(AJE) for further polishing(Order ID:HBKG9NHW). Our editing certificate is below.

Comment # 2: Authors declared that there were several meta-analysis in this field and there were published before 2011 and that is the reason they performed this study. Only two included studies published after 2011, do they have great impact on the results and evidences?! What is the benefit of your study?

Response: Thank you for your concern and your question is crucial to our study. For the first question. We do had some inconsistent conclusions compared with previous study. Our study drew a conclusion that IFNs were more effective than NAs. This result was not inconsistent with Yang’s study^[1]^, which proved IFNs and NAs were equally effective at causing proteinuria remission. What’s more, Wang et al^[2]^ performed a meta-analysis and declared that NA monotherapy could protect renal function and preventing Scr elevation. However, our results didn’t show significant improvement in renal function, especially under treatment of NAs.

The meta-analysis published recently didn’t give suggestions on details of therapy, such as treatment for different ages of patients, antiviral drug selection or treatment duration. Our meta-analysis mainly focus on these problems, this is our advantage. Our study aim to help clinicians dealing with complicated clinical situations. Laying the foundation for a standardized treatment guidlines which is personalized and precise for the current therapeutic regimen are lacking.

Reference:

[1] Yang Y , Ma Y , [Chen DP](https://www.ncbi.nlm.nih.gov/pubmed/?term=Chen DP[Author]&cauthor=true&cauthor_uid=27598699), [Zhuo L](https://www.ncbi.nlm.nih.gov/pubmed/?term=Zhuo L[Author]&cauthor=true&cauthor_uid=27598699), [Li WG](https://www.ncbi.nlm.nih.gov/pubmed/?term=Li WG[Author]&cauthor=true&cauthor_uid=27598699). A Meta-Analysis of Antiviral Therapy for Hepatitis B Virus-Associated Membranous Nephropathy. Plos One. 2016; 11(9):e0160437.
[2] Wang WN, Wu MY, Ma FZ, [Sun T](https://www.ncbi.nlm.nih.gov/pubmed/?term=Sun T[Author]&cauthor=true&cauthor_uid=26636326), [Xu ZG](https://www.ncbi.nlm.nih.gov/pubmed/?term=Xu ZG[Author]&cauthor=true&cauthor_uid=26636326). Meta-analysis of the efficacy and safety of nucleotide/nucleoside analog monotherapy for hepatitis B virus-associated glomerulonephritis. Clinical Nephrology. 2016; 85(1):21-29.

Comment # 3: Mention the exact PICOS of the study at the end of the introduction section.
Response: Thank you for your advice and we have mentioned the exact PICOS of our study at the end of the introduction section on page4, line3-5.
Comment # 4: Mention the exact time you performed your search, in the methods section .
Response: Thank you for your advice and we have mentioned the exact time of our search on page, line15.
Comment # 5: Provide search strategy for at least one database.

Response:We are very sorry our negligence of this. We have provided search strategy of Pubmed in supporting information.

Comment # 6: Why did you searched PUBMED and MEDLINE?? When you are searching PUBMED you are searching MEDLINE, too.
Response: It’s really true as you said, but we searched MEDLINE through OVID platform. We could get more recent literatures through PUBMED detabase, and MEDLINE(Ovid Online) could complement the missing literatures.

### Comment # 7: Provide more information about covering grey literature. How did you cover this and which conferences did you check. Response: Thank you for your queation. The title and abstract of conference papers could be covered by Pubmed database, Medline,and Web of Knowledge, otherwise, we also serched in [official](../../../../C:/Program%20Files%20(x86)/Youdao/Dict/8.5.3.0/resultui/html/index.html#/javascript:;) [website](../../../../C:/Program%20Files%20(x86)/Youdao/Dict/8.5.3.0/resultui/html/index.html#/javascript:;)s of societies. We checked Asian Pacific Congress of Nephrology,[Australia and New Zealand Society of Nephrology](http://www.baidu.com/link?url=Sntjj-C9AOZ5Ojb7yrp92IbjlhsTW_xaDf3Umq8IasnCKiDU536hXV2Bf5mXbnKy), conferences held by American Urological Association, International Society of Nephrology, [American Society of Nephrology](http://www.baidu.com/link?url=pHf689j3f6uYQ7no-7ZDLvXtHcmuY_NJfIHgQ7-WZCZPop4QXQKcnLeniXiOIakq) and [European Association of Urology](http://www.baidu.com/link?url=J4SqalX1hPOc0U31o4SlVxjxpv2_rPrZyvB1I0DpSd3).

Comment # 8: Did you consider any specific language in the search? Four of seven included studies are from China.
Response: We felt sorry that because of language restrictions, only English literature were searched. Trials in our meta-analysis were conducted in Asian countries and vastly limited to China. This geographic concentration is probably the reason of a high prevalence of HBV infection in these countries. As a result, our conclusions may not apply to European or American patients. We analyzed these limitations in discussion on page 20,line9-11.

Comment # 9:Write “Eligibility criteria” section in an organized way. It is really confusing.

Response:We totally agree with your suggestion and revised eligibility criteria section on page 5,line1-8.

Comment # 10: Mention the outcome you expected to find in the included articles, in the “Eligibility criteria” section.

Response: It is really true as reviewer suggested, but we actually didn’t set the outcomes as accordance while screening of the literature, we only prescribe limits to patients and interventions. We really except to find remission of proteinuria, clearence of HBeAg or eGFR as outcomes, but if the trials didn’t cover these indicators, we still included this study. Hoping get your understanding.

Comment # 11:In “Data extraction” section, mention the exact variables you extracted from the studies. Do not use “So on”.

Response: We totally agree with your suggestion, we revised this part on page 5,line22-23.

Comment # 12: Did you pay attention to funding source of the studies? You did not mention this.
Response: Thanks for your suggestion. We looked up the funding source of including studies. We found that all studies mentioned funding source except Panomsak’s study. The funding providers of six studies were mostly hospital, medical school and medical council, no pharmaceutical firm were mentioned. In this case, we considered founding sources won’t cause basis on results. Strictly speaking, Panomsak’s study may induce slight basis. While, Panomsak’s study was a retrospective study and various kinds of drugs were used, we don’t think this trial could inclined to any drugs.

Comment # 13: Third line of “Statistical analysis” section, the sentence “In the absence of...” can be deleted. You have discussed this further.

Response: We feel sorry that it was writing mistake, we deleted the sentence“In the absence of...”.

Comment # 14: You included seven studies. Four from China, two from other parts of Asia and one from Africa. How do you discuss this distribution?

Response: It's really true as you suggested, this geographic concentration is probably the reason of a high prevalence of HBV infection in these countries. As a result, our conclusions concerning the efficacy and safety of antiviral treatments may not apply to European or American patients. We have discussed this limitation in discussion section on page 20,line 9-12.

Comment # 15: “Subgroup analysis of association between efficacy and study types” part should be totally revised. First, discuss the heterogeneity results and then report the analysis results.

Response: We totally agree with you and we have totally revised this part on page 11, line1-7.
Comment # 16:. In “Subgroup analysis of association between efficacy and study types” section, heterogeneity of CCTs was reported twice in the 8th and 11th line with different statistics. Please rewrite this part and make it clear if they are different variables.

Response:We feel sorry for our carelessness. In our resubmitted manuscript, the first heterogeneity of CCTs is deleted. Thanks for your correction.
Comment # 17: In the second line of “Effects on eGFR” section, replace “anti-virus therapy” with “anti-viral therapy”.

Response: We have made correction according to the reviewer’s comments. We replaced “anti-virus therapy” with “anti-viral therapy” on page13, line 5.

Comment # 18:In the fifth line of “Effects on eGFR” section, authors declared “it seems that NAs has therapeutic effect on eGFR”. However, the reported confidence interval includes zero. Please clarify this part.

Response: We are very sorry our incorrect writing, NAs has no therapeutic effect on eGFR according to our results. We deleted sentence “In trials treated with NAs, it seems that NAs has therapeutic effect on eGFR (MD=1.06, 95% CI: -1.28−3.39) and showed no statistical significance with I2=0%, P=1.00.”
Comment # 19: The discussion part is written like introduction section. Study results should be discussed and compared with other studies. Discussion section needs an overall revision.

Response: We sincerely appreciate this comments and we have revised discussion part already. We deleted the first paragraph about clinical status  of HBV-GN and our benifits. We analysed our results from the very beginning in this part. Second, we added comparison of other studies versus of our study, which were colored in red. Third, we discussed our conclusions and limitations. We appreciate for your warm work earnestly, and hope that the correction will meet with approval.

Thank you very much for giving us an opportunity to revise our manuscript, We have tried our best to revise our manuscript  according to the comments. We would like to thank the reviewers for their helpful comments and hope that we have now produced a more balance and better account of our work. We trust that the revised manuscript is acceptable for publication. Looking forward to hear from you.

Thank you and best regards.

Yours sincerely,

Baohui Fu and Yue Ji

Corresponding author:

Name: Hongtao Yang

Emile: [tjpdyht@163.com](mailto:tjpdyht@163.com)
